# 741. *Ehrlichia chaffeensis* Induced Hemophgocytic lymphohistiocytosis: A Descriptive Case Series

**DOI:** 10.1093/ofid/ofab466.938

**Published:** 2021-12-04

**Authors:** jeffrey lin, Hanine El Haddad, Ayman Qasrawi, Gerhard Hildebrandt

**Affiliations:** University of Kentucky, Lexington, Kentucky

## Abstract

**Background:**

Hemophagocytic lymphohistiocytosis (HLH) secondary to tick borne illnesses is rarely reported. Clinical signs and symptoms of tick borne illnesses and HLH might overlap with fever, cytopenias and increased liver enzymes being common. We describe findings from case series of ehrlichiosis induced HLH.

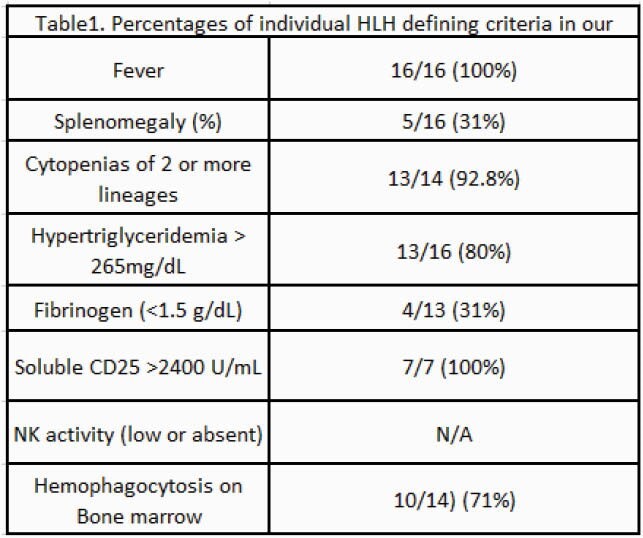

**Methods:**

We reviewed patients with ICD-10 codes corresponding to a diagnosis of HLH or macrophage activation syndrome (MAS) at University of University of Kentucky Medical Center between January 2008 and April 2020. Inpatients who were >18 years of age without known immune compromise were included. 4 cases with confirmed underlying ehrlichiosis were identified at our institution. We searched PubMed for English-language articles containing the terms “ Hemophagocytic lymphohistiocytosis “ and “infection” or “tick borne” or “Ehrlichia”. Data on patient demographics, clinical signs and symptoms, laboratory data such as ferritin, platelet count, Il-2, NK cell activity, and outcomes were collected.

**Results:**

We identified 16 cases of ehrlichiosis (1 had a coinfection with Rocky Mountain Spotted fever). Eleven out of 6 (68%) were male, median age was 58. All patients were febrile and thrombocytopenic on presentation and 8/14 (57%) were neutropenic. All had elevated ferritin (mean 36187 ng/mL, range 860 – more than 100000). CNS involvement was reported in 4 patients with a positive CSF Ehrlichia *chaffensis* PCR. All patients met at least 5 2004-HLH defining criteria and 10/14 (71%) patients had evidence of hemophagocytosis on bone marrow biopsy (table 1). Fourteen out of 15 (93%) patients received doxycycline and 9/15 (60%) received steroids +/- etoposide. Mortality for Ehrlichia induced HLH was 12.5%, significantly lower than that reported for all secondary HLH mortality (45%).

**Conclusion:**

This review highlights the importance of considering Ehrilichiosis as a cause of HLH in endemic areas particularly as clinical signs and symptoms of the 2 entities overlap. While overall mortality rate due to HLH is elevated, Ehrlichia induced HLH seems to have a much favorable prognosis with prompt institution antimicrobial treatment. Additional prognostic factors that correlate with a more severe course dictate need for immunosuppressive treatment need to be further elucidated.

**Disclosures:**

**Gerhard Hildebrandt, MD**, **Bayer, Scotts-Miracle, Charlottes Webb CWBHF, Almmune Therapeutics Inc AIMT, Medical PPTYS TR Inc. MPW, Caretrust Reit Inc CTRE, ANGI Homeservices Inc** (Shareholder)**Bristil-Myers Squibb/Medarex, Crispr therapeutics, IDEXX Laboratories, Johnson & Johnson, Pfizer, Procter & Gamble, Vertex** (Shareholder)**Falk Foundation, Incyte, Takeda** (Other Financial or Material Support, Travel, Accommodations, Expenses)**Jazz Pharmaceuticals, Incyte, Morphosys, Alexion Pharmaceuticals, Karyopharm Therapeutics, Seattle Genetics** (Consultant)**Jazz Pharmaceuticals, Pharmacyclics, Incyte, AstraZeneca** (Grant/Research Support)**Novartis, Insys Therapeutics, Abbvie, GW Pharmaceuticals, Cardinal Health, Clovis Oncology, Cellectis, CVS Health, Celgene, Bluebird Bio** (Shareholder)

